# Comparison of Efficacy of Cannabinoids versus Commercial Oral Care Products in Reducing Bacterial Content from Dental Plaque: A Preliminary Observation

**DOI:** 10.7759/cureus.6809

**Published:** 2020-01-29

**Authors:** Veronica Stahl, Kumar Vasudevan

**Affiliations:** 1 Dentistry, Euro Dent Belgium, Mortsel, BEL; 2 Genetics, Cannibite, Antwerp, BEL

**Keywords:** cannabinoids, dental plaque, antibacterial, oral care products, personalized dental care

## Abstract

Background

Dental plaque is a complex biofilm that gets formed on the teeth and acts as a reservoir of different microbes. It is the root cause for the occurrence of several dental problems and diseases, including cavities, bad breath, bleeding gums, tooth decay, and tooth loss. Therefore, it should be regularly removed using suitable oral care aids.

Objectives

The present study compared the efficacy of oral care products and cannabinoids in reducing the bacterial content of dental plaques.

Methods

Sixty adults aged 18 to 45 years were categorized into six groups based on the Dutch periodontal screening index. Dental plaques of the adults were collected using paro-toothpick sticks and spread on two Petri dishes, each with four divisions. On Petri dish-A, cannabidiol (CBD), cannabichromene (CBC), cannabinol (CBN), and cannabigerol (CBG) were used, and on Petri dish-B, cannabigerolic acid (CBGA), Oral B, Colgate, and Cannabite F (a toothpaste formulation of pomegranate and algae) were used. The Petri dishes were sealed and incubated, followed by counting the number of colonies.

Results

By evaluating the colony count of the dental bacteria isolated from six groups, it was found that cannabinoids were more effective in reducing the bacterial colony count in dental plaques as compared to the well-established synthetic oral care products such as Oral B and Colgate.

Conclusion

Cannabinoids have the potential to be used as an effective antibacterial agent against dental plaque-associated bacteria. Moreover, it provides a safer alternative for synthetic antibiotics to reduce the development of drug resistance.

## Introduction

Dental plaque refers to the complex biofilm that acts as a reservoir of several microbes. It can be defined as “the soft deposit that forms the biofilm adhering to the tooth surface or other hard surfaces in the oral cavity, including removable and fixed restorations.” Dental plaque is formed owing to the deposition of a combination of saliva, foods, and fluids on the tooth surface. The dental plaque formed on the tooth surface and gum line includes thousands of bacteria that convert food residues into acids, eventually leading to the initiation of dental diseases such as dental caries, gingivitis, and periodontal diseases. Periodontitis or gum disease is a global public health problem that affects millions of people each year and is the most common cause of tooth loss in adults [1]. It is a gum infection that affects the soft and hard tissues supporting the teeth.

As dental plaque is the primary cause associated with several dental diseases, it should be regularly removed using different kinds of oral care aids, including mechanical aids such as toothbrushes, interdental floss, and interdental brushes, and chemical aids such as mouthwashes and dentifrices. These are considered good oral hygiene aids in improving and promoting an individual’s oral health [2]. There exist several approaches for removing dental plaques; however, as periodontal diseases are caused by bacterial infection of soft and hard tissues anchoring the teeth, antimicrobial treatment serves as an effective adjunct for plaque control and, in turn, improves the inflamed tissues of gums and bones.

Arrays of antimicrobial agents are available in the market, such as chlorhexidine digluconate, which is the golden standard for an antimicrobial agent and others such as Colgate and Oral B. Along with the commercially available agents for reducing bacterial content of dental plaque, several natural herbal extracts, such as pomegranate, algae, triphala, tulsipatra, neem, aloe vera, and cinnamon, have been reported to be effective against dental plaque bacteria [3-9]. Similarly, cannabinoids extracted from cannabis has been reported to have potential antimicrobial properties against both gram-positive and gram-negative bacterial species [10-12]. However, at least to our knowledge, the efficiency of cannabinoids in inhibiting the growth of dental plaque associated bacteria has not been reported so far.

Cannabis, popularly known as marijuana, is derived from the plant Cannabis sativa, which has gained enormous popularity recently owing to its multiple benefits, especially in the field of cosmetics and pharmacology [13]. Cannabinoids are a group of secondary metabolites produced by the small glands called trichomes present on the surface of the plant and act on the cellular cannabinoid receptors [14]. Approximately, more than 100 kinds of cannabinoids are produced by the plant [15].

Cannabinoids are divided into three groups on the basis of their source: endogenous or endocannabinoids (produced in humans and animals), synthetic (produced in the laboratory), and phytocannabinoids (uniquely from the cannabis plant) [16]. A few examples of cannabinoids are Δ-8-THC, cannabidiol (CBD), cannabinol (CBN), cannabigerol (CBG), and cannabichromene (CBC) [17]. These cannabinoids present in Cannabis are known for their antibacterial properties. It has been found that some cannabinoids, such as CBD, CBC, CBG, CBN, and ∆9-tetrahydrocannabinol (THC), exert antibacterial activity against a variety of methicillin-resistant *Staphylococcus aureus* (MRSA) strains [17]. Cannabidiol has been identified as a component of hemp oil that is effective against gram-positive bacteria and yeast [18]. Wasim et al. (1995) tested ethanol and petroleum extracts of cannabis leaves against different microorganisms. The results showed that the extracts have strong inhibitory effects on both gram-positive bacteria (*Bacillus subtilis*, *Bacillus pumilus*, *S. aureus*,and *Micrococcus flavus*) and gram-negative bacteria (*Proteus vulgaris* and *Bordetella bronchiseptica*) [19].

To the best of our knowledge, no such study has been published that compares the efficiency of cannabinoids with that of oral care products against dental bacteria. Our study is the first of its kind conducted to compare the efficacy of well-established commercial oral care products and cannabinoids in reducing the bacterial content of the dental plaque. Reducing the bacterial content could significantly decrease and prevent gum diseases that have become a huge global burden owing to their direct relation with systemic diseases. Here we report a preliminary observatory study on effect of cannabinoids on reducing the bacterial content of dental plaque.

## Materials and methods

Study population

A randomized controlled trial was conducted from January 2019 to March 2019 to assess the efficacy of cannabinoids in comparison to the efficacy of commercial oral care products in reducing the bacterial content of the dental plaque. The study protocol was reviewed and cleared by the Ethics Committee of the Institutional Review Board (AZ Groeninge Kortrijk; Belgium). The study protocol and the purpose were explained to the participants, and consent from each participant was obtained before the start of the study.

A total of 60 healthy adults, aged 18 to 45 years, were recruited for the study from EuroDent clinic, Belgium. The candidates satisfying the following criteria were selected for the study: (a) presence of a minimum number of teeth (seven), including one molar, (b) absence of dentures, (c) no recent history of antimicrobial therapy or other drug therapy, including immunosuppressives, and (d) no history of diabetes.

The participants were categorized into six groups (10 participants in each group) on the basis of Dutch periodontal screening index (DPSI) as follows: 0, perfect gum and no bleeding; 1, inflammation and bleeding of gum (gingivitis); 2, conditions of category 1 and chalk hardened dental plaque; (-3), conditions of category 2 with bone involvement (periodontitis); (+3) conditions of (-3) with recessions of gum and root exposure; and 4, conditions of category (+3) with severe bone resorption and high tooth mobility.

Sample collection

**Figure 1:**
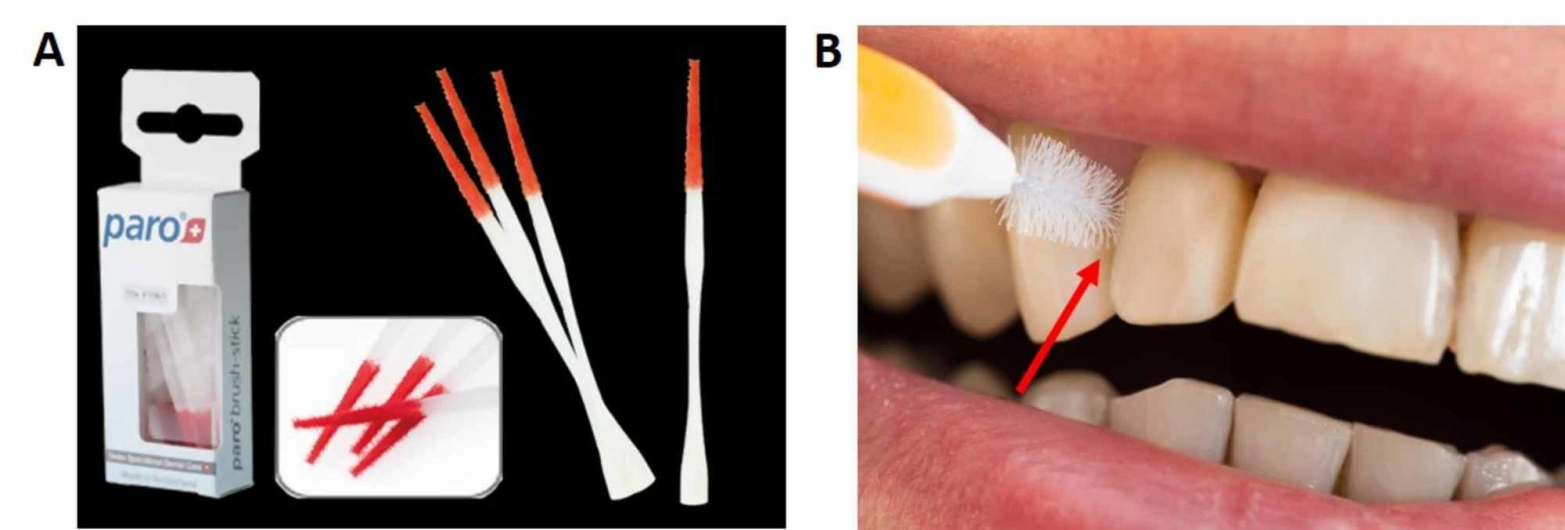
Dental plaque sampling (A) Paro-toothpick sticks consisting of red velvet on the active part. (B) Dental plaque sampling spot (indicated by arrow) from the interdental space.

Prior to plaque sampling, saliva on the tooth surface was removed by water spray, and the sampling target area was dried with cotton. Plaque samples were collected from interdental spaces using a paro-toothpick stick consisting of red velvet on the active part that could easily pick up the dental plaque (Figure 1). The collected plaque samples were directly spread on two Petri dishes (marked as A and B) consisting of lysogeny broth agar and pre-treated with test components as described below.

In vitro assay

Four divisions were made on the Petri dishes A and B. On each section, cannabinoid (12.5%) or toothpaste (undiluted) was spread/streaked on the surface of the agar plate using microbrush applicator. On Petri dish A, CBD, CBC, CBN, and CBG were used, and on Petri dish B, cannabigerolic acid (CBGA), Oral B, Colgate, and Cannabite F (a toothpaste formulation of pomegranate and algae) were used. The dental plaque sample was spread/streaked over the same area of the agar plate pre-treated with cannabinoids or toothpaste. The two Petri dishes were sealed with paraffin film and incubated at 37°C for 24 hours. After 24 hours, colony counting was performed in automated colony counter (acolyte3-Synbiosis).

Data analysis

The colony count values from 10 individuals of each of the six groups were respectively combined to obtain a cumulative value for each group against each product tested. Statistical analysis was not performed due to lack of replicates. Similarly, statistical analysis was not performed by combining the data from 10 individuals in each group due to the fact that the oral microflora greatly differ in every individual.

## Results

We evaluated the colony count of dental plaque samples of 10 candidates from each of the six research groups on exposure to cannabinoids or toothpastes. As an example, we describe here the bacterial colony count results of a single representative candidate from each of the six research groups (Table 1). In group DPSI 0, the maximum number of colonies was found with the Oral B treatment, whereas the minimum number of colonies was present in the CBN treatment. In group DPSI 1, the maximum number of colonies was found in Oral B treatment and the minimum number was present in the CBC treatment. In group DPSI 2, the maximum number of colonies was found in the Colgate treatment and the minimum number was found in the CBC treatment. In the DPSI (-3) group, the maximum number of colonies was found in the Oral B treatment and the minimum number in the CBGA treatment. In the DPSI (+3) group, the maximum number of colonies was found in the Oral B treatment and the minimum number was present in the CBN treatment. In the DPSI 4 group, the maximum number of colonies was present in the Oral B treatment and the minimum number was found in the CBN treatment (Table 1). In all six research groups studied, the maximum bacterial growth was observed in Oral B, Colgate, and Cannabite F treatments. The colony count in cannabinoid treatments were all significantly lower than that recorded in any of the toothpaste tested. Among the cannabinoids tested, CBN and CBC were effective in several research groups (Table 1). The complete colony count data of individual candidates of all research groups studied are available in Table 2.

**Table 1:** Bacterial colony count of single candidate dental plaque sample from six DPSI research groups against cannabinoids CBGA, cannabigerolic acid; CBN, cannabinol; CBG, cannabigerol; CBD, cannabidiol; CBC, cannabichromene and Cannabite F, formulation of pomegranate and algae.

Treatments (on LB plate)	Bacterial colony count of candidate dental plaque sample from six research groups						
	DPSI 0	DPSI 1	DPSI 2	DPSI (–3)	DPSI (+3)	DPSI 4	
CBGA	13	4	9	3	14	5	
CBN	4	8	3	7	2	1	
CBG	20	12	8	4	8	9	
CBD	7	6	8	6	5	8	
CBC	11	3	2	11	9	2	
Oral B	35	38	25	31	38	34	
Colgate	12	27	34	25	32	27	
Cannabite F	14	32	18	21	21	24	

**Table 2:** Comparison of efficacy of cannabinoids vs commercial oral care products in reducing the bacterial content from dental plaque DPSI, Dutch periodontal screening index; CBGA, cannabigerolic acid; CBN, cannabinol; CBG, cannabigerol; CBD, cannabidiol; CBC, cannabichromene and Cannabite F, formulation of pomegranate and algae.

DPSI group / Treatments	Mean colony count										Total	Average
	P1	P2	P3	P4	P5	P6	P7	P8	P9	P10		
DPSI 0												
CBGA	13	5	13	7	8	14	18	4	17	16	115.00	11.5
CBN	4	14	8	15	16	9	5	12	8	13	104.00	10.4
CBG	20	25	18	19	14	11	14	9	4	12	146.00	14.6
CBD	7	12	4	20	12	21	9	15	13	3	116.00	11.6
CBC	11	11	12	9	6	17	17	18	9	9	119.00	11.9
Oral b	35	4	9	34	12	24	24	24	18	22	206.00	20.6
Colgate	12	40	25	12	28	12	12	35	19	34	229.00	22.9
Cannabite F	14	12	18	17	15	18	32	18	7	19	170.00	17
DPSI 1												
CBGA	4	11	9	12	8	9	7	12	4	5	81.00	8.1
CBN	8	4	3	4	5	5	5	2	8	6	50.00	5
CBG	12	8	9	7	3	4	1	5	12	9	70.00	7
CBD	6	14	5	6	10	2	8	7	6	3	67.00	6.7
CBC	3	2	6	2	7	11	4	11	3	1	50.00	5
Oral b	38	23	24	27	34	21	24	33	38	23	285.00	28.5
Colgate	27	28	28	21	31	28	28	38	27	24	280.00	28
Cannabite F	32	18	19	24	27	18	23	27	32	25	245.00	24.5
DPSI 2												
CBGA	9	2	14	12	14	3	8	6	11	3	82.00	8.2
CBN	3	7	2	6	4	9	12	12	7	12	74.00	7.4
CBG	8	5	10	9	7	4	7	9	5	4	68.00	6.8
CBD	8	11	5	3	13	12	9	14	3	7	85.00	8.5
CBC	2	4	3	5	11	7	1	5	1	10	49.00	4.9
Oral b	25	18	19	28	32	18	21	17	21	34	233.00	23.3
Colgate	34	25	27	21	28	21	28	24	38	23	269.00	26.9
Cannabite F	18	21	24	29	25	15	15	14	19	24	204.00	20.4
DPSI -3												
CBGA	3	6	7	6	11	5	12	5	13	8	76.00	7.6
CBN	7	3	1	2	4	2	7	9	4	4	43.00	4.3
CBG	4	9	9	13	9	8	3	3	7	9	74.00	7.4
CBD	6	1	2	5	3	2	1	1	8	2	31.00	3.1
CBC	11	4	5	4	1	6	4	4	2	4	45.00	4.5
Oral b	31	24	23	25	14	32	22	35	29	38	273.00	27.3
Colgate	25	37	17	19	17	28	29	38	32	35	277.00	27.7
Cannabite F	21	18	19	17	13	17	17	18	18	22	180.00	18
DPSI +3												
CBGA	14	3	8	4	5	9	3	6	8	7	67.00	6.7
CBN	2	6	4	7	9	6	7	8	3	1	53.00	5.3
CBG	8	4	2	2	4	2	2	2	6	3	35.00	3.5
CBD	5	14	6	8	3	4	9	7	2	4	62.00	6.2
CBC	9	7	7	2	1	7	1	1	1	2	38.00	3.8
Oral b	38	25	26	27	27	33	24	37	34	27	298.00	29.8
Colgate	32	36	17	31	22	29	31	34	37	37	306.00	30.6
Cannabite F	21	18	14	18	14	16	18	19	16	19	173.00	17.3
DPSI 4												
CBGA	5	4	5	7	8	7	9	4	8	6	63.00	6.3
CBN	1	3	1	4	5	5	7	3	3	4	36.00	3.6
CBG	9	5	9	2	2	3	9	2	7	7	55.00	5.5
CBD	8	9	8	8	4	8	3	5	2	3	58.00	5.8
CBC	2	4	2	1	1	1	1	1	1	1	15.00	1.5
Oral b	34	32	34	28	27	24	29	32	27	31	298.00	29.8
Colgate	27	38	27	34	21	26	25	38	34	32	302.00	30.2
Cannabite F	24	25	24	24	32	21	18	19	21	28	236.00	23.6

**Figure 2:**
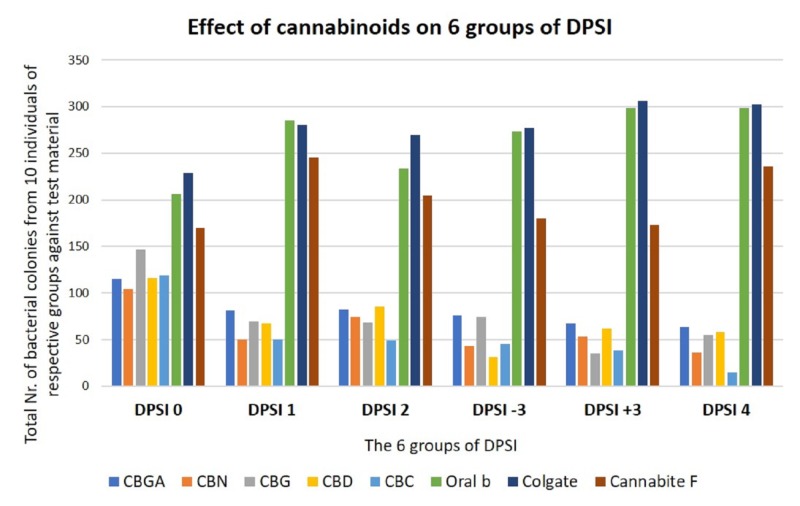
Comparison of six research groups with respect to bacterial colony count DPSI, Dutch periodontal screening index; CBGA, cannabigerolic acid; CBN, cannabinol; CBG, cannabigerol; CBD, cannabidiol; CBC, cannabichromene and Cannabite F, formulation of pomegranate and algae.

We pooled the colony count data of all 10 candidates from each research group respectively to study the overall outcome. As expected, the bacterial colony count was much higher in Colgate, Oral B treatments and Cannabite F treatment, whereas significantly less colony count was observed in all cannabinoid treatments (Table 2) (Figure 2). Similarly, on average, CBC and CBN were effective against dental plaque bacteria from more than one research group (Figure 2). In addition to pooled data, it is interesting to observe the individual data of candidates because in any given DPSI group, the efficiency of cannabinoids varied from individual to individual as seen in Table 2.

## Discussion

*Cannabis sativa* L. (*C. sativa L.)* is an herbaceous plant that belongs to the family Cannabinaceae. It is known by several names worldwide, such as marijuana in America; bhang, ganja, and charas in India; kif in North Africa; dogga in South Africa; and djomba or liamba in Central Africa and Brazil. It is believed to be originated from Central Asia, and it is one of the oldest psychoactive plants known [20,21].

*C. sativa L. *contains several psychoactive compounds known as cannabinoids. It has been reported that there are more than 100 types of cannabinoids present in plants and they have been used for their medicinal, recreational, and spiritual properties for over 5000 years [15,22]. In India, it has been used to induce anesthesia and as an anti-phlegmatic agent before the 10th century B.C. In the 20th century B.C, it was used for treating sore eyes in Egypt [23]. In the northeastern part of India, the plant is used to treat several diseases such as allergies, burns, cuts and wounds, inflammation, leprosy, leucoderma, scabies, smallpox, and sexually transmitted diseases [24].

Among the several cannabinoids, THC is found to be the predominant and the most psychoactive component [16]. Furthermore, these cannabinoids are found to contain several antibacterial properties. The antibacterial feature is attributed primarily to the presence of Δ9-tetrahydrocannabinol (Δ9-THC) and CBD. THC, the primary psychoactive constituent, mediates its pharmacological effects mainly through G protein-coupled central cannabinoid (CB1) receptors present in the brain. Significant binding to the receptors in the cerebellum, hippocampus, basal ganglia, and cerebral cortex correlates with the cannabinoid effects on pain, cognition, memory, movement, and endocrine functions [25,26].

The endocannabinoid (EC), anandamide (AEA), and arachidonoyl serine (AraS) exert antibacterial properties against MRSA strains. Moreover, they have the potential to modify the bacterial membrane and prevent biofilm formation [10]. *C. sativa* extracts exert antimicrobial activity on gram-positive bacteria, such as *B. subtilis*, *B. pumilus*, *S. aureus*, *M. flavus*; gram-negative bacteria such as *P. vulgaris*, *B. bronchioseptica*, *Pectobacterium carotovorum*, and *Pseudomonas savastanoi*, as well as certain fungi such as *Aspergillus niger *and *Candida albicans* [19,27].

The first evidence of interference of *C. sativa*-derived products in the bacterial signal transduction systems by a synthetic cannabinoid HU-210 was reported by Soni et al. in 2015. They concluded that the synthetic cannabinoid HU-210 has an inhibitory effect on quorum sensing (QS) and QS-dependent properties, such as bioluminescence, biofilm formation, and swimming motility of* Vibrio harveyi*, without affecting its growth [28]. Feldman et al. (2018) demonstrated that the tested compounds (AEA in particular) could impair the pathogenicity of MRSA by inhibiting their ability to form biofilm, reducing the metabolic activity of mature biofilm, and modifying the bacterial cell surface characteristics without killing the bacteria. They concluded that ECs and EC-like compounds may serve as a natural line of defense against MRSA or other antibiotic-resistant bacteria. Such cannabinoids, owing to their anti-biofilm action, could be a promising alternative to antibiotic therapeutics against biofilm-associated MRSA infections [10].

In the present study, we compared the efficacy of oral care products and cannabinoids in reducing the bacterial content of dental plaques. Dental plaque is a structurally organized biofilm containing millions of bacteria and is responsible for several oral diseases such as gingivitis, periodontitis, and dental caries. The majority of microorganisms forming the biofilm of the dental plaque are gram-positive bacteria, such as *Streptococcus mutans*, followed by gram-negative bacteria and several other anaerobes such as *Fusobacterium* and *Actinobacteria*. In our study, cannabinoids were found to be more effective in reducing the colony count of the bacterial strains as compared to the well-established synthetic oral care products such as Oral B or Colgate. In the 1950s, the topical preparations from *C. sativa* were found to contain antiseptic properties against several oral cavities as well as skin lesions [29].

Ali et al. (2012) studied the effect of* C. sativa L*. seed oil as well as petroleum ether and methanol extracts of the whole plant on two gram (+) bacteria (*B. subtilis* and *S. aureus*) and two gram (-) bacteria (*Escherichia coli* and *Pseudomonas aeruginosa*). The cannabis seed oil displayed a strong antibacterial activity with a zone of growth inhibition (21-28 mm) against *B. subtilis* and *S. aureus* and moderate activity (15 mm) against *E. coli* and *P. aeruginosa *(16 mm) [30].

In the present study, we compared the antibacterial efficacy of synthetic cannabinoids and commercial oral care products. To our knowledge, this is the first report of its kind involving in vitro assay of cannabinoids against dental plaque samples directly collected from patients. The advantage of such an approach is that the samples represent natural and collective oral biofilm diversity (that are culturable) from each candidate, in contrast to conventional testing against one or two lab grown strains of bacteria. Most of the published reports have used one or few pure bacterial isolates to study the antimicrobial activity against oral bacteria. Although such pure bacterial isolates involve common and major pathogenic oral bacteria, it is important to consider the diversity of oral microflora between individuals and their biological relevance on oral health. Therefore, sampling that involve total bacterial content in such study will bring added value to the data. 

However, the present study had certain limitations that should be addressed. First, the sample size of our study was 60, which is less for a clinical trial. Second, we included both normal and gingivitis and periodontitis patients. Hence, more randomized controlled trials should be conducted for a longer duration consisting of a larger sample size and only with periodontitis patients for the exact comparison of the results and assessment of long-term effects of synthetic cannabinoids on oral health care. Moreover, this was a preliminary observatory study involving simple testing methodology without replicates. 

Oral health is an integral part of general health and well-being; however, it is neglected by most of the people. It could be improved using oral health care products such as an appropriate toothpaste, toothbrush, tongue cleaner, mouthwash, and floss. The selection of appropriate oral health care products could play a critical role in improving oral health and in preventing dental diseases. However, the most common problem faced by people is the difficulty in selecting the right oral care product. As shown in the present study, even the most commonly used commercial toothpastes lack the efficacy to completely reduce bacterial count from the mouth. The bacterial composition of oral biofilm varies from person to person. As shown in the present study, even the efficiency of cannabinoids may vary from individual to individual owing to the nature of individual oral biofilm. Hence, a personalized approach would be appropriate to identify the best formulation of oral care that fits into the requirement and nature of biofilm of an individual. Moreover, a scheduled repetition of oral care hygiene procedures is a must to obtain the desired results as it takes a lifetime of care to achieve a healthy mouth.

## Conclusions

Although commercially available oral care products are considerably effective in maintaining the oral hygiene of the average population, our study found that cannabinoids are substantially effective in reducing the colony count of the bacterial strains of the dental plaque as compared to the well-established synthetic oral care products such as Oral B and Colgate. In addition, our results suggest that the efficiency of cannabinoids could vary from individual to individual plausibly owing to the microbial diversity of oral biofilms. More detailed analysis on effect of cannabinoids on oral microflora will be studied to understand and validate this observatory study by involving larger sample size, replicates and proper methodology. We believe that our study opens up the possibilities of developing personalized next-generation oral care products based on cannabinoids. 

## Appendices

Authors' Contributions

VS designed and conducted the project. VS analyzed the data and wrote the manuscript. KV involved in writing and editing the manuscript.
